# Comprehensive Transcriptome Profiling of Antioxidant Activities by Glutathione in Human HepG2 Cells

**DOI:** 10.3390/molecules29051090

**Published:** 2024-02-29

**Authors:** Yoshiaki Uchida, Farhana Ferdousi, Shinya Takahashi, Hiroko Isoda

**Affiliations:** 1 Research and Development Division, Mitsubishi Corporation Life Sciences Ltd., 1-1-3 Yurakucho, Tokyo 100-0006, Japan; yoshiaki.uchida@mcls-ltd.com; 2 Institute of Life and Environmental Sciences, University of Tsukuba, Tsukuba 305-8572, Japantakahashi.shinya.fp@u.tsukuba.ac.jp (S.T.); 3 Alliance for Research on the Mediterranean and North Africa (ARENA), University of Tsukuba, Tsukuba 305-8572, Japan; 4 Open Innovation Laboratory for Food and Medicinal Resource Engineering (FoodMed-OIL), National Institute of Advanced Industrial Science and Technology (AIST), Tsukuba 305-8577, Japan

**Keywords:** glutathione, antioxidative stress, reactive oxygen species, DNA microarray, HepG2 cell

## Abstract

Glutathione (GSH) has long been recognised for its antioxidant and detoxifying effects on the liver. The hepatoprotective effect of GSH involves the activation of antioxidative systems such as NRF2; however, details of the mechanisms remain limited. A comparative analysis of the biological events regulated by GSH under physiological and oxidative stress conditions has also not been reported. In this study, DNA microarray analysis was performed with four experiment arms including Control, GSH, hydrogen peroxide (HP), and GSH + HP treatment groups. The GSH-treated group exhibited a significant upregulation of genes clustered in cell proliferation, growth, and differentiation, particularly those related to MAPK, when compared with the Control group. Additionally, liver functions such as alcohol and cholesterol metabolic processes were significantly upregulated. On the other hand, in the HP-induced oxidative stress condition, GSH (GSH + HP group) demonstrated a significant activation of cell proliferation, cell cycle, and various signalling pathways (including TGFβ, MAPK, PI3K/AKT, and HIF-1) in comparison to the HP group. Furthermore, several disease-related pathways, such as chemical carcinogenesis–reactive oxygen species and fibrosis, were significantly downregulated in the GSH + HP group compared to the HP group. Collectively, our study provides a comprehensive analysis of the effects of GSH under both physiological and oxidative stress conditions. Our study provides essential insights to direct the utilisation of GSH as a supplement in the management of conditions associated with oxidative stress.

## 1. Introduction

Reactive oxygen species (ROS) are the endogenous byproducts of cellular oxidative metabolism. They encompass various chemically reactive molecules such as superoxide anions, hydrogen peroxide (HP), and hydroxyl radicals. They play integral roles in numerous cellular functions, including cell growth and death, signal transduction, immune responses, and defence against microorganisms [1]. They can interact with different biological targets within cells and modify them owing to their inherent reactivity. However, excessive ROS accumulation can lead to various diseases, including cancer, neurodegenerative disorders, diabetes, and cardiovascular diseases [2,3,4,5].

The liver is a vital organ involved in metabolism and detoxification and is particularly susceptible to the detrimental effects of excessive ROS [6]. Reactive oxygen species are predominantly generated within the mitochondria and the endoplasmic reticulum of hepatocytes by cytochrome P450 enzymes [7]. Oxidative stress occurs when there is an imbalance between oxidants and antioxidants, resulting in structural and functional abnormalities in hepatocytic proteins, lipids, and DNA [8]. Chronic liver diseases such as nonalcoholic fatty liver disease (NAFLD), alcoholic liver disease, viral hepatitis, and fibrosis consistently exhibit an increase in oxidative stress [9,10,11,12]. Therefore, protection against excessive ROS production is a key strategy to maintain optimal liver function and promote overall health.

Glutathione (GSH) is a tripeptide composed of cysteine, glutamic acid, and glycine [13]. It is a naturally occurring antioxidant found in various dietary sources, such as spinach, avocados, asparagus, and okra. It is abundant in nearly all human cells, with particularly high concentrations in the liver, where it plays a crucial role in various cellular processes, such as proliferation and differentiation. It has garnered significant attention for its potent antioxidant properties since it acts as a detoxifier of free radicals and helps regulate ROS balance [14,15]. Numerous studies show the effectiveness of GSH in neurones, skin, and other tissues [16,17]. Notably, GSH has promising effects in liver diseases [18].

In vitro studies have elucidated the mechanisms underlying the therapeutic effects of GSH. GSH treatment activates the NRF2 signalling pathway in various cell lines, including liver cells [19,20,21]. Furthermore, several reports highlight the regulation of upstream NRF2 signalling pathways such as MAPK and PI3K/AKT by GSH in neurones or macrophages [20,21,22]. These findings support the antioxidant properties of GSH. However, the hepatoprotective mechanisms of GSH are partially studied, and there is a lack of comprehensive whole-molecular studies of the hepatoprotective mechanisms, including up- and downstream of NRF2. Moreover, our understanding of the precise comparison of GSH’s effects under physiological and oxidative stress conditions remains limited.

A global gene expression analysis was conducted in this study, using DNA microarrays to elucidate the underlying molecular mechanisms of supplemented GSH and provide a comprehensive overview of the effects. HepG2 cells were utilised to assess the transcriptome-wide changes induced by supplemented GSH under normal physiological and HP-induced oxidative stress conditions since they are widely used as an in vitro liver model. In the present study, HP is utilised to induce the oxidative stress since it is produced from almost all source of oxidative stress and widely used for oxidative research.

## 2. Results

### 2.1. Glutathione Protects against HP-Induced Oxidative Stress in HepG2 Cells

**Figure 1:**
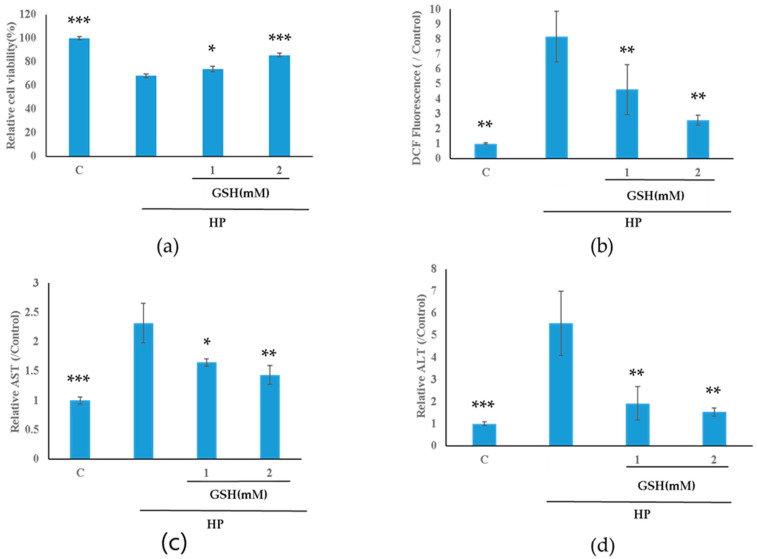
Protective effect of glutathione (GSH) on oxidative stress in HepG2 cells. HepG2 cells were pretreated with 1 or 2 mM GSH for 24 h and were exposed to 2 mM of hydrogen peroxide (HP) for 4 h. All data are presented as the mean ± standard deviation (SD). Comparison with the HP-treated group was carried out using one-way analysis of variance (ANOVA) followed by Dunnett’s test (* *p* < 0.05, ** *p* < 0.01, *** *p* < 0.001). (**a**) Cell viability analysed using the 3-(4,5-dimethylthiazol-2-yl)-2,5-diphenyltetrazolium bromide (MTT) assay. (**b**) Reactive oxygen species assay analysis. (**c**,**d**) Liver function markers (aspartate aminotransferase (AST) and alanine aminotransferase (ALT)) analysed by enzyme-linked immunosorbent assay (ELISA).

Cell viability was evaluated following exposure to HP and pretreatment with various GSH concentrations. Exposure to 2 mM HP significantly reduced cell viability compared to the Control group (Figure 1a). However, pretreatment with GSH mitigated this HP-induced decrease in cell viability in a dose-dependent manner (Figure 1a). Exposure to HP significantly elevated intracellular ROS production measured as DCF fluorescence in HepG2 cells. However, this effect was significantly reversed in a dose-dependent manner in the GSH-pretreated groups (Figure 1b).

Excessive ROS levels typically prompt hepatocytes to increase the expression of hepatic markers, such as aspartate aminotransferase (AST) and alanine aminotransferase (ALT), which are commonly used indicators to assess hepatic health. Enzyme-linked immunoassays (ELISAs) showed that treatment with GSH dose-dependently reduced AST and ALT levels compared to those in the group exposed to HP alone (Figure 1c,d). These findings indicated that GSH protected HepG2 cells against oxidative stress.

### 2.2. Characteristics of Gene Expression Profiling in GSH-, HP-, and GSH + HP-Treated HepG2 Cells Compared to Untreated Control Cells

HepG2 cells were subjected to different treatments, including Control, GSH, HP, and GSH + HP. Three specific comparisons were conducted between these groups to ensure a comprehensive analysis: GSH vs. Control (representing the effect of GSH under normal conditions), HP vs. Control (representing oxidative stress induction), and GSH + HP vs. HP (representing the preventive effect of GSH against oxidative stress).

**Figure 2:**
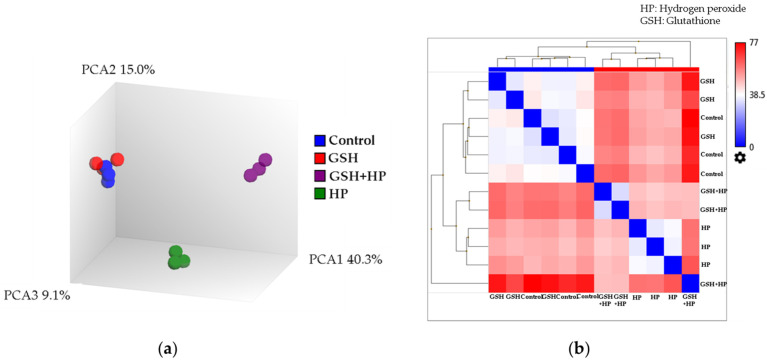
HepG2 cells were pretreated with 1 mM of GSH for 24 h and exposed to 2 mM of HP for 4 h. Three comparison groups were set to analyse the effects of GSH (HP vs. Control: oxidative stress, GSH vs. Control: normal condition, and GSH + HP vs. HP: prevention of oxidative stress). (**a**) Principal component analysis. (**b**) Euclidean distance matrix showing the distance between the samples. The tSNE algorithm was used for the nonlinear dimensional reduction approach.

The principal component analysis (PCA) plot showed that the Control and the GSH-treated groups were similar (Figure 2a). This indicated a high degree of similarity between their gene expression profiles. In contrast, the GSH-treated and HP-treated groups exhibited a linear noncorrelation with each other and with the Control and GSH groups. This suggested that GSH pretreatment modulated the gene expression profile of HP-treated cells in a distinct manner.

The corresponding distance map was derived from the correlation analysis (Figure 2b). This distance matrix was computed using the Euclidean metric and provided insights into the relationships between the samples. It was consistent with the PCA plot and demonstrated that the Control and GSH samples exhibited a closer relationship and lower distance values. In contrast, the HP-induced samples (GSH-treated and untreated) displayed greater distances from the Control and GSH samples. This indicated a greater dissimilarity in their gene expression profiles.

### 2.3. Differential Gene Expression in GSH-, HP-, and GSH + HP-Treated HepG2 Cells Compared to Untreated Control Cells

**Figure 3:**
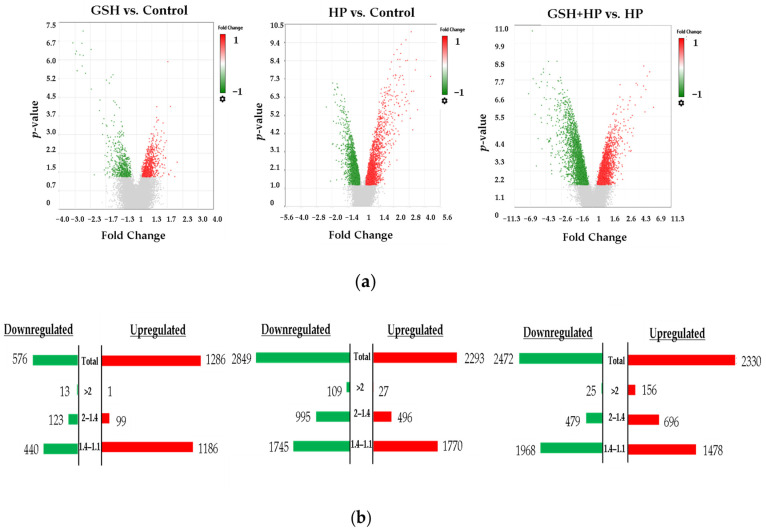
Characterisation of gene expression profiles in GSH-treated HepG2 cells. (**a**) Volcano plots showing the differentially expressed genes (DEGs) between GSH vs. Control, GSH + HP vs. HP or HP vs. Control comparisons. The y axis shows the −log10 *p*-value, and the x axis shows the fold change. Red dots and bars display the upregulated genes, and the green dots and bars display the downregulated genes. (**b**) The butterfly charts show the distribution of fold changes of DEGs. The bars represent the number of DEGs.

The differential gene expression analysis is shown as volcano plots and butterfly charts (Figure 3). In the volcano plots, upregulated genes are represented by red dots, whereas green dots denote downregulated genes. Butterfly charts present the distribution of fold change (FC) in gene expression for each comparison.

Microarray analyses using the Clariom S Assay Human cartridge identified 21,448 genes. Overall, 1862 differentially expressed genes (DEGs) were identified between the GSH and Control groups (1286 upregulated and 576 downregulated).

There was a total of 5142 genes showing DEGs in the HP group compared with the Control group (2293 upregulated and 2849 downregulated).

A total of 4802 DEGs were identified comparing GSH + HP with HP (2330 upregulated and 2472 downregulated).

The HP group displayed the highest number of downregulated DEGs compared with the Control group among the comparisons made, with an FC > 1.1. Conversely, the GSH + HP group exhibited the highest number of upregulated DEGs compared with the HP group, with an FC > 1.1.

### 2.4. Gene Ontology (GO) and Kyoto Encyclopaedia of Genes and Genomes (KEGG) Pathway Enriched by the GSH vs. Control, HP vs. Control, and GSH + HP- vs. HP-Treated HepG2 Cells

**Figure 4:**
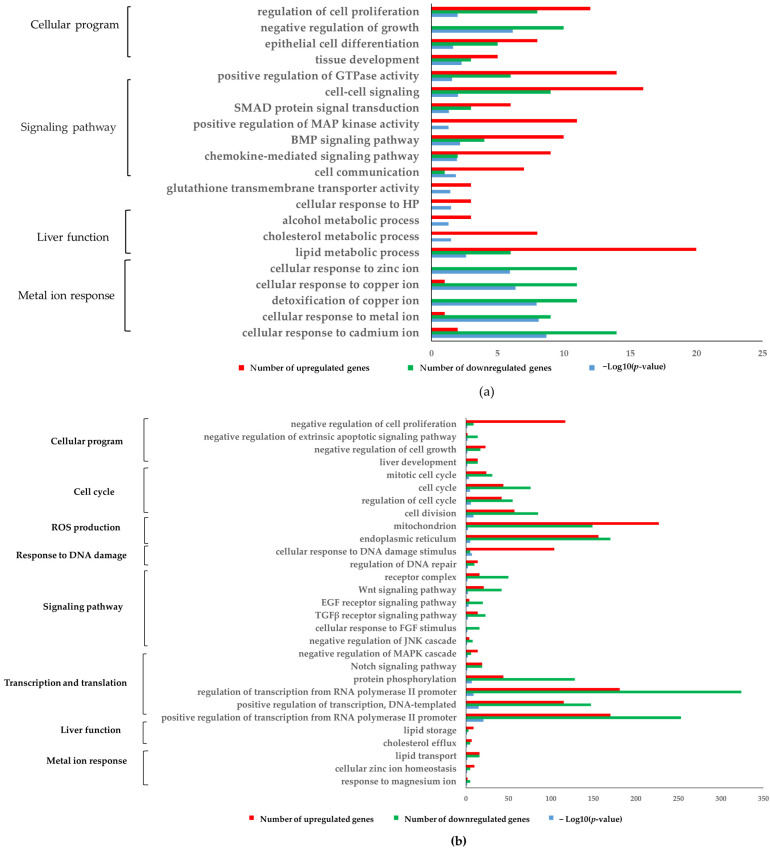

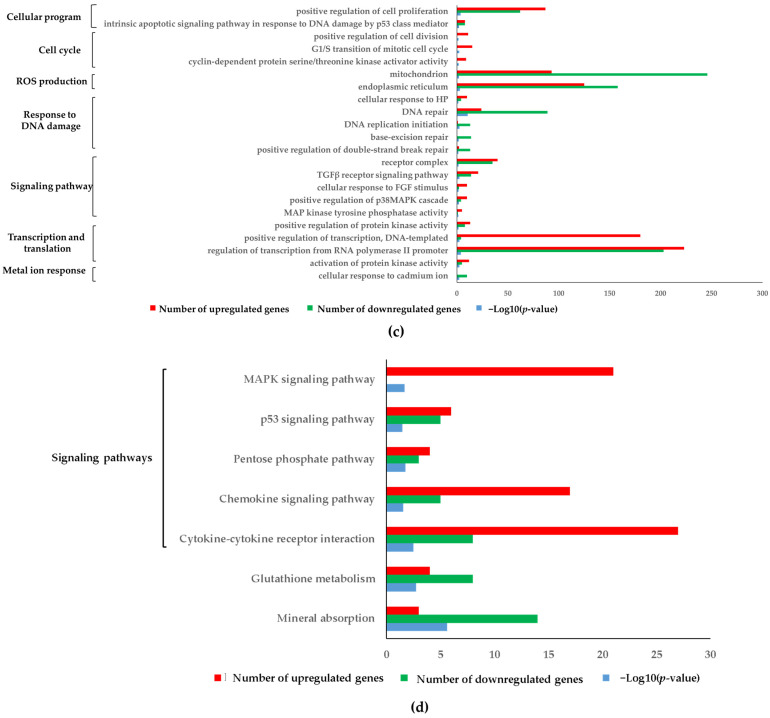

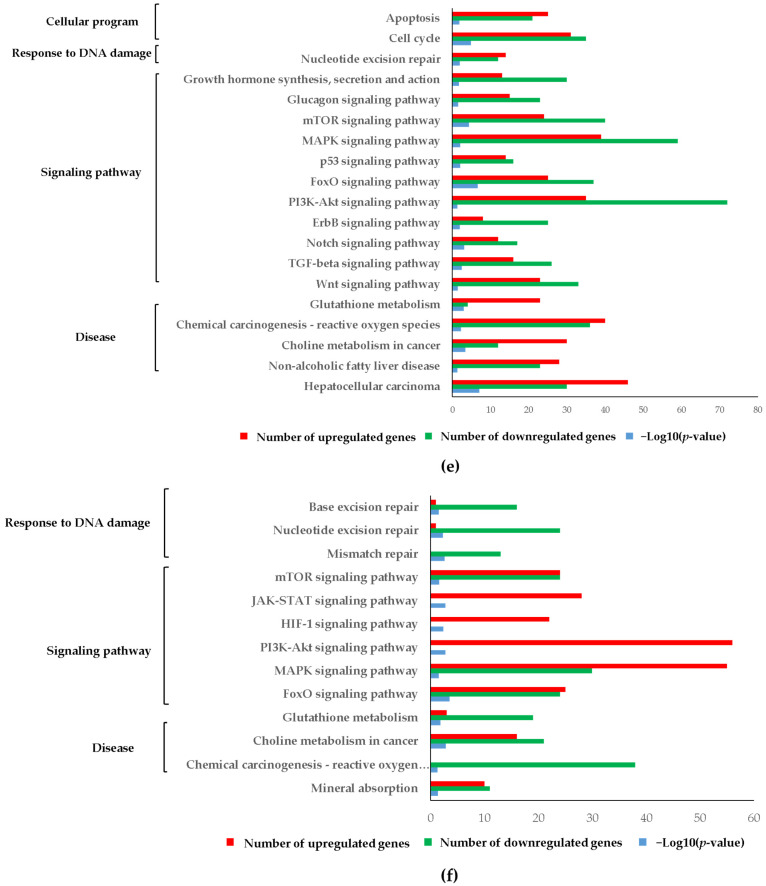
Overview of Gene ontology (GO) and Kyoto Encyclopaedia of Genes and Genomes (KEGG) analysis. The difference in the GOs and significantly enriched pathways between the three conditions are visualised. The red, green, and blue colours represent the number of upregulated genes, the number of downregulated genes, and the –log10 (*p*-value), respectively. Significantly enriched GO terms by (**a**) GSH vs. Control, (**b**) HP vs. Control, and (**c**) GSH + HP vs. HP. Significantly enriched KEGG pathways in the (**d**) GSH vs. Control group comparison, (**e**) HP vs. Control group comparison, and (**f**) GSH + HP vs. HP group comparison.

A Gene Ontology analysis was conducted to investigate the main biological domains regulated in each comparison. The GSH vs. Control comparison showed the activation of cell proliferation, differentiation, and tissue development (Figure 4a). The HP vs. Control group comparison showed the inactivation of cell proliferation and growth and the activation of the apoptotic signalling pathway (Figure 4b). Meanwhile, GSH + HP and HP groups activated cell proliferation (Figure 4c). The expression of cell cycle-related genes was predominantly downregulated in the HP vs. Control comparison and upregulated in the GSH + HP vs. HP comparison. Mitochondria and the endoplasmic reticulum are strongly related to ROS production. A comparison of HP vs. Control showed an upregulation of mitochondria, while the opposite result was observed in GSH + HP vs. HP group comparison. The endoplasmic reticulum was predominantly downregulated in the HP vs. Control and GSH + HP vs. HP groups. The expression of DNA damage-related genes was predominantly upregulated in the HP group compared to the Control group, whereas it was downregulated in the GSH + HP group compared to the HP group. The upregulated genes in the GSH-treated group were enriched in signalling pathways: MAPK, BMP, and SMAD were activated. Furthermore, a comparison of GSH + HP vs. HP group showed the activation of TGFβ, FGF, and MAPK. In contrast, the downregulated genes were enriched in the Wnt, EGF, TGFβ, FGF, JNK, MAPK, and Notch signalling pathways when comparing HP vs. Control group. The GSH transmembrane transporter-related genes were mostly upregulated in the GSH vs. Control group. The cellular response to HP was activated in both of the GSH-treated groups. Transcription- and translation-related GO terms were predominantly downregulated in the HP vs. Control condition, including protein phosphorylation, regulation of transcription from the RNA polymerase II promoter, and positive regulation of DNA-templated transcription. Conversely, comparison of the GSH + HP vs. HP group showed upregulated transcription- and translation-related GO terms. Moreover, there was activation of liver function-related GO terms, such as alcohol, cholesterol, and lipid metabolism in the GSH vs. Control group comparison. The liver function-associated GO terms (including lipid storage and cholesterol efflux) were differentially expressed after comparing HP and Control groups. Moreover, metal ion response-related terms were inactivated in both of the GSH-treated groups. Cellular responses to zinc-, copper-, metal-, and cadmium ion-related genes were mostly downregulated in the GSH group. A comparison of GSH + HP vs. the HP group showed an inactivation of the cellular response to Cd ions. Meanwhile, the HP vs. Control group comparison showed activation of cellular zinc ion homeostasis and inactivation of the response to magnesium ions.

Significantly enriched KEGG of Genes and Genomes pathways were identified using the Database for Annotation, Visualisation, and Integrated Discovery (DAVID). Apoptosis-related genes were predominantly upregulated in the HP vs. Control group, and cell cycle genes were downregulated (Figure 4e). Nucleotide excision repair-related genes were upregulated to a greater degree in HP than in the Control. In contrast, base excision repair, nucleotide excision repair, and mismatch repair were downregulated in the GSH + HP vs. HP group comparison. The signalling pathway-associated GO terms were mostly upregulated in the GSH vs. Control group and GSH + HP vs. HP group comparisons. Meanwhile, comparison of HP vs. the Control group showed opposite trends to the those of the GSH-treated groups (Figure 4d,f). The KEGGs affected by comparing GSH vs. Control group were the MAPK, p53, pentose phosphate, and chemokine signalling pathways. A comparison of GSH + HP vs. HP group showed activation of the JAK-STAT, HIF-1, PI3K/AKT, MAPK, and FoxO signalling pathways. KEGGs affected by HP vs. Control included growth hormone synthesis, secretion and action, glucagon, mTOR, MAPK, p53, FoxO, PI3K/AKT, ErbB, Notch, TGF, and the Wnt signalling pathway. Glutathione metabolism showed opposite trends between GSH and HP. Glutathione metabolism-related genes were predominantly downregulated in the GSH vs. Control and GSH + HP groups. In contrast, the HP vs. Control conditions showed an upregulation. In addition, downregulated mineral absorption-related genes were more abundant than upregulated genes in the GSH vs. Control and GSH + HP vs. HP groups. The HP vs. Control and GSH + HP vs. HP groups showed the opposite trend regarding liver-related diseases. Cancer-related terms such as chemical carcinogenesis, ROS, choline metabolism in cancer, and hepatocellular carcinoma were activated in the HP vs. Control group. Nonalcoholic fatty liver disease-associated genes were also predominantly upregulated in the HP group compared with the Control. Meanwhile, the GSH + HP showed the inactivation of choline metabolism in cancer and chemical carcinogenesis related to ROS.

### 2.5. Alternation of HP-Induced Oxidative Stress by GSH

**Figure 5:**
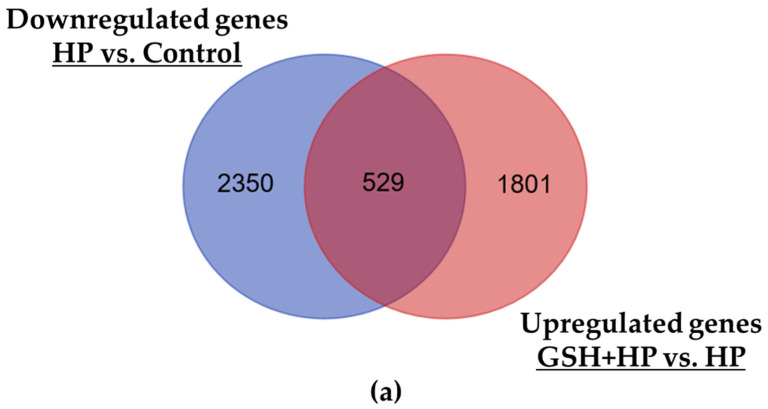

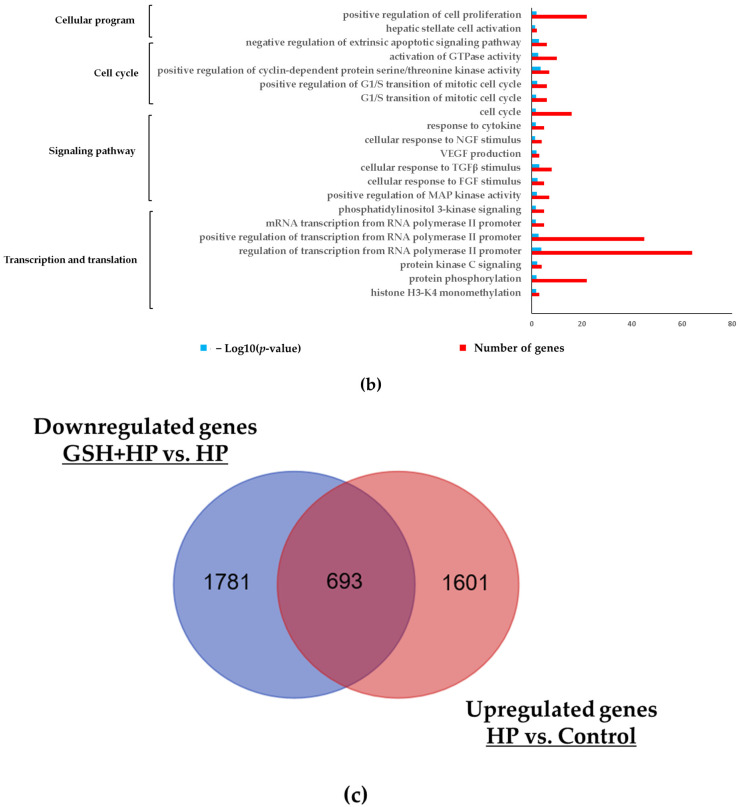

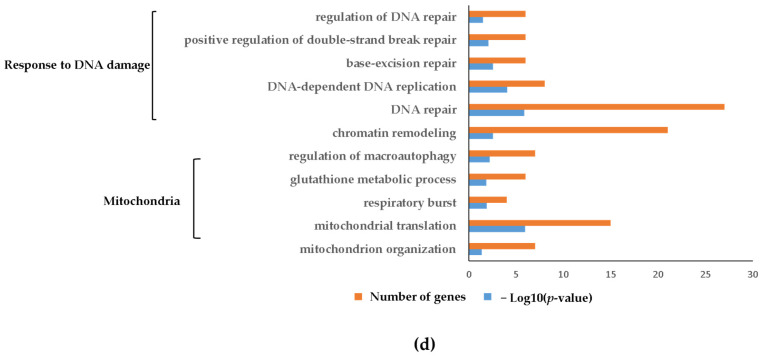
Venn diagrams showing the numbers of unique and common DEGs between different comparison groups. Bar graphs showing significantly enriched GO terms by the common gene sets between two comparison conditions. (**a**) The blue circle displays downregulated DEGs in HP vs. Control group. The red circle displays upregulated DEGs in GSH + HP vs. HP group. (**b**) Significantly enriched GO terms in common sets of HP vs. Control group (down) and GSH + HP vs. HP group (up). (**c**) The blue circle displays downregulated DEGs in GSH + HP vs. HP group. The red circle displays upregulated DEGs in HP vs. Control group. (**d**) Significantly enriched GO terms in common sets of HP vs. Control group (up) and GSH + HP vs. HP group (down).

Subsequently, we examined the effect of GSH on HP-induced oxidative stress. Venn diagrams showed a common and unique DEG set between the HP vs. Control and GSH + HP vs. HP group comparisons (Figure 5a,c). A total of 529 genes that were significantly downregulated in HP-induced cells (HP vs. Control group) were significantly upregulated in GSH-pretreated cells (GSH + HP vs. HP group). Conversely, 693 genes that showed significant upregulation in HP-induced cells compared to the Control group (HP vs. Control) were significantly downregulated in the GSH-pretreated group compared to the HP group (GSH + HP vs. HP). The 529 common genes between HP vs. Control (down) and GSH + HP vs. HP (up) showed significantly enriched GO-related genes to cellular programmes, such as positive regulation of cell proliferation, hepatic stellate cell activation, and negative regulation of the extrinsic apoptotic signalling pathway (Figure 5b). Cell cycle-associated GO terms included activation of GTPase activity, positive regulation of cyclin-dependent protein serine/threonine kinase activity, and positive regulation of G1/S transition of the mitotic cell cycle and cell cycle. Moreover, signalling pathways such as cytokine, NGF, VEGF, TGFβ, FGF, MAPK, and phosphatidylinositol 3-kinase signalling were activated. Positive regulation of transcription from the RNA polymerase II promoter, protein kinase C signalling, protein phosphorylation, and histone H3-K4 monomethylation were activated. A total of 693 common genes between HP vs. Control (down) and GSH + HP vs. HP (up) showed significantly enriched GO terms related to response to DNA damage, such as regulation of DNA repair, positive regulation of double-strand break repair, base excision repair, DNA-dependent DNA replication, and chromatin remodelling (Figure 5d). Macroautophagy regulation was also activated. The mitochondria-related GO terms included glutathione metabolic process, respiratory burst, mitochondrial translation, and mitochondrial organisation.

### 2.6. Detection of Gene Clusters and Functional Modules

**Figure 6:**
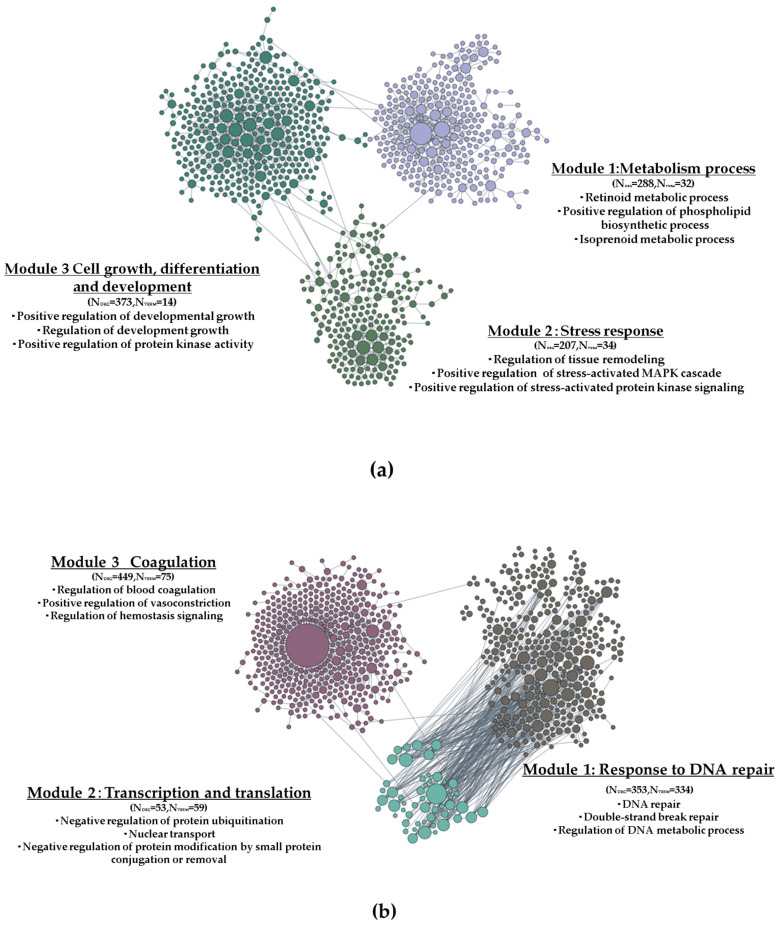
Functionally clustered modules of DEGs determined from the HumanBase public database. Significantly enriched GO terms of each module are presented. Significance was determined by Fisher’s exact tests followed by Benjamini–Hochberg corrections. Term: Number of enriched GO terms. The colors of dots showing the different modules. (**a**) Top modules of GSH + HP vs. HP by upregulated DEGs. (**b**) Top modules of GSH + HP vs. HP by downregulated DEGs.

Functionally clustered genes were generated using the HumanBase public database and analysed using a liver-specific network. Functional clusters allowed us to interpret the tight biological networks between these genes. We analysed the upregulated and downregulated DEGs separately under the conditions of GSH + HP vs. HP. Two thousand three hundred and thirty upregulated DEGs generated from the GSH + HP vs. HP group comparison revealed three modules (Figure 6a). Module 1 contained 288 DEGs with 32 significantly enriched biological process (BP) terms related to metabolic processes, such as retinoid metabolic processes, positive regulation of phospholipid biosynthetic processes, and isoprenoid metabolic processes. Module 2 contained 207 DEGs related to stress responses, such as the regulation of tissue remodelling, positive regulation of the stress-activated MAPK cascade, and positive regulation of stress-activated protein kinase signalling. Module 3 contained 373 DEGs related to cellular programmes such as positive regulation of developmental growth, regulation of developmental growth, and positive regulation of protein kinase activity. In contrast, 2472 downregulated DEGs from the GSH + HP vs. HP condition were analysed (Figure 6b). Module 1 contained 353 DEGs with 334 significantly enriched BP terms related to DNA repair responses, such as DNA repair, double-strand break repair, and regulation of DNA metabolic processes. Module 2 contained 53 DEGs related to transcription and translation, including negative regulation of protein ubiquitination and nuclear transport and negative regulation of protein modification by small protein conjugation or removal. Module 3 contained 449 DEGs related to coagulation, such as the regulation of blood coagulation, positive regulation of vasoconstriction, and regulation of haemostasis signalling.

### 2.7. Common and Unique DEGs in Control, GSH, HP, and GSH + HP Groups in HepG2 Cells

**Figure 7:**
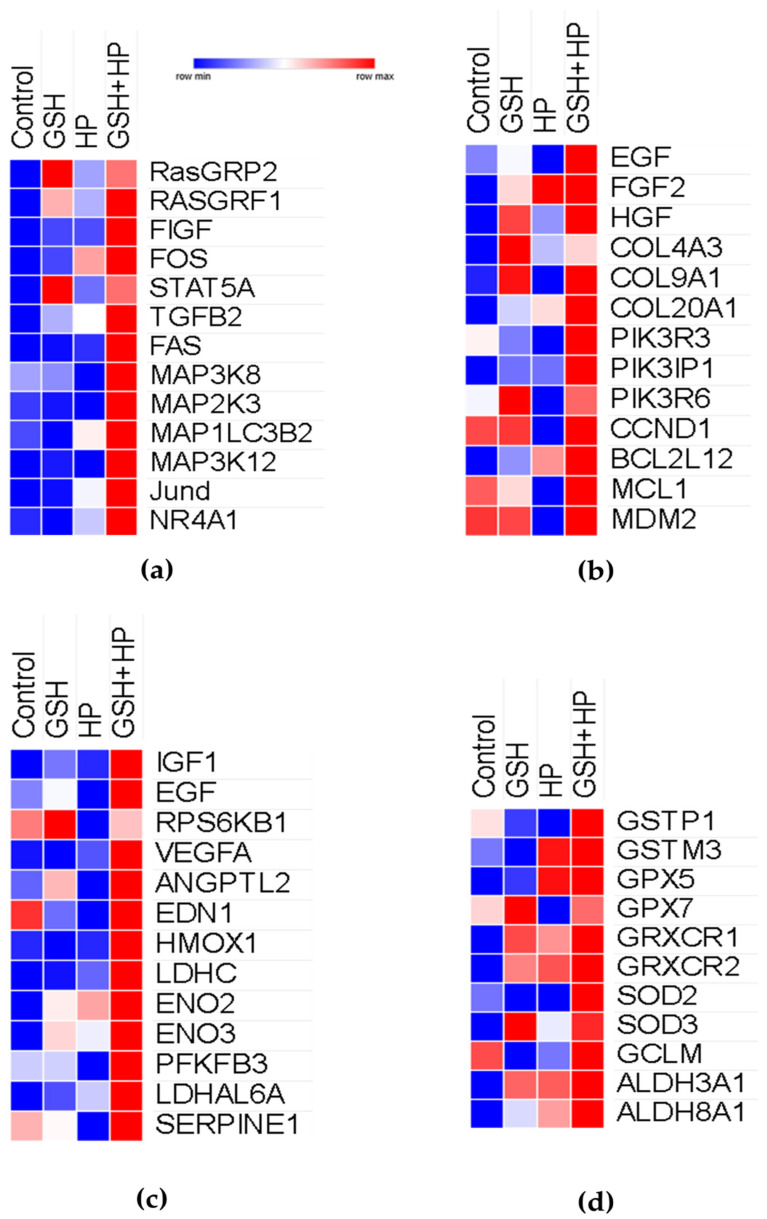
Heatmaps showing expression pattern of genes related to stress response signalling pathways. (**a**) MAPK-signalling-related genes. (**b**) PI3K/AKT signalling-related genes. (**c**) HIF-signalling-related genes. (**d**) NRF2-signalling-related genes.

**Figure 8:**
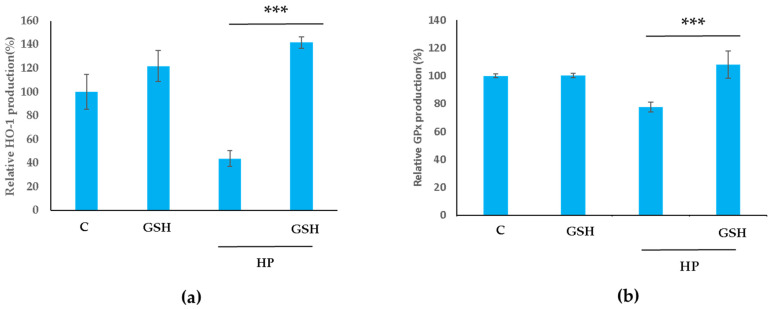
Antioxidant-related enzyme’s expression in HepG2 cells. HepG2 cells were pretreated with 1 mM GSH for 24 h and were exposed to 2 mM of hydrogen peroxide (HP) for 4 h. All data are presented as the mean ± standard deviation (SD). Comparison with the Control or HP-treated and GSH-treated groups were carried out by t test (*** *p* < 0.001). (**a**) The protein expression level of HO-1 analysed by enzyme-linked immunosorbent assay (ELISA). (**b**) The protein expression level of GPx analysed by ELISA.

Finally, heat maps summarise the DEGs related to antioxidant survival systems activated by GSH treatment in the present study and in previous publications (Figure 7). A few genes were activated more than those in the Control group, while the GSH + HP group activated the MAPK signalling pathway (Figure 7a). The PI3K/AKT, GSH, and GSH + HP groups were strongly activated (Figure 7B). Commonly upregulated genes were *HGF*, *COL9A1*, *PIK3R6*, *CCND1*, *MCL1*, and *MDM2*. HIF-1 signalling pathway-related genes were especially upregulated in the GSH + HP group compared to the HP group (Figure 7C). NRF2 signalling pathway-associated genes were upregulated in the GSH, HP, and GSH + HP groups (Figure 7D). The commonly upregulated genes between the GSH and GSH + HP groups were *GPX7*, *GRXCR1*, and *GRXCR2* and *SOD3* and *ALDH3A1*. In addion, we performed ELISA to check the protein expression level of antioxidant-related enzymes such as HO-1 and GPx (Figure 8). In the GSH + HP group, HO-1 and GPx were significantly expressed compared with the HP group.

## 3. Discussion

We have presented a comprehensive analysis of transcriptome-wide changes induced by GSH in HepG2 cells under normal and oxidative stress conditions. We explored the effects of GSH on cellular pathways and their molecular responses to oxidative stress to shed light on its potential implications in promoting liver health and mitigating oxidative stress-related liver disorders.

Glutathione is an antioxidant peptide naturally formed in the liver [13]. It has important functions in the liver since this organ is particularly susceptible to ROS [23]. Previous studies demonstrate the efficacy of GSH against liver diseases, such as NAFLD [18,24,25]. However, endogenous GSH levels naturally decrease with age [26]. Additionally, the human body poorly absorbs dietary GSH since cooking and storage conditions can decrease the amount of GSH in food [27]. Therefore, there was an increasing trend of GSH supplementation. The hepatoprotective effects of GSH and the mechanism by which it activates NRF2 are known. However, the comprehensive molecular mechanism, including NRF2’s upstream and downstream in the liver are poorly studied. Moreover, there is no comparative analysis of GSH’s effects under physiological and oxidative stress conditions.

This study showed that the GSH treatment group significantly activated cell proliferation, differentiation, development, and several signalling pathways (including BMP) under normal conditions that are well-known differentiation-related signals (Figure 4a). Moreover, the GSH treatment group significantly activated essential liver function-related GO terms, such as alcohol metabolism and cholesterol storage. A previous study reported that endogenous GSH has a crucial role in cellular processes, such as proliferation and differentiation in the human body [28]. Moreover, GSH is related to alcohol metabolism [29]. Therefore, our result demonstrated the potential of GSH treatment for activating cellular programmes such as growth and enhancing liver functions under normal conditions.

Glutathione is a widely researched antioxidant compound owing to its protective effects against oxidative damage in some cell lines [20,21,22]. This study showed that GSH has a protective effect against oxidative stress (Figure 1a–d). Additionally, GSH activated the MAPK, PI3K/AKT, and HIF-1 signalling pathways against oxidative stress (Figure 4c,f). The GSH-treated group without oxidative stress significantly activated MAPK signalling compared to the Control group (Figure 4a). In contrast, MAPK and PI3K/AKT signalling were inactivated in the oxidative stress-induced groups (Figure 4b,e). Moreover, Nrf2 signalling was activated, especially in the GSH + HP group (Figure 7d). Nrf2 is the primary regulator as a transcriptional factor of antioxidant genes that induces antioxidant response elements [30]. NRF2 is phosphorylated by several kinases, including the MAPK and PI3K/AKT signalling pathways [31,32]. The MAPK and PI3K/AKT pathways play crucial roles in cell proliferation and survival. In addition, HIF-1 is a master regulator of cellular oxygen sensing, cell proliferation, and survival, and HIF-1 activation is mediated via MAPK or NRF2 [33,34]. Previous studies demonstrate the activation of NRF2 and one of these kinases following GSH treatment in macrophage and neurone cell lines [20,21]. This study elucidated the details of the hepatoprotective mechanisms of GSH involving the activation of NRF2 and related signalling pathways including MAPK, PI3K/AKT, and HIF-1 in liver cells. The elucidation of these findings at the molecular level owing to comprehensive microarray analysis is significant for the study of GSH’s hepatoprotective mechanism. Furthermore, oxidative stress increases the risk of several diseases including cancer and NAFLD [35,36]. In our study, the HP-treated groups (but not the GSH + HP group) showed activation of functional events, including carcinoma and NAFLD (Figure 4e). Moreover, tumour growth and metastasis depend on angiogenesis [32], and angiogenesis-related terms were inactivated comparing GSH + HP vs. HP group (Figure 6B). These results indicate that GSH activates the antioxidant system and attenuates the risk of HP-induced liver diseases.

We elucidated the alteration of the GSH-specific effect against HP-induced oxidative stress by common and unique DEG sets between the HP vs. Control and GSH + HP vs. HP groups (Figure 5a,c). Negative regulation of apoptotic signals, proliferation, and cell cycle-related GO terms was significantly activated among the common genes from HP vs. Control group (down) and GSH + HP vs. HP group (up) (Figure 5b). Furthermore, several signalling pathways (including NGF, VEGF, FGF, TGFβ, and MAPK) were activated. These results indicate that GSH stimulates growth factor-related signalling pathways and MAPK and protects against oxidative stress by promoting proliferation. In contrast, the common genes from HP vs. Control group (up) and GSH + HP vs. HP group (down) showed activation in response to DNA damage-related GO terms. We hypothesised that GSH protected HepG2 cells from HP, resulting in reduced DNA damage. Moreover, mitochondria-related GO terms were activated in the HP vs. Control (top) and GSH + HP vs. HP (bottom) groups. In addition, the GSH-treated groups showed inactivation of GSH metabolism (Figure 4e,f) and activation of GSH transmembrane transporter activity (Figure 4a). These results suggest that GSH treatment supplies sufficient amounts of GSH to the cells, and there is no longer a need to produce it.

This study showed the comparison of GSH’s effect under normal and oxidative conditions. Under normal conditions, GSH activated cellular programmes, such as growth, and enhanced liver functions. Meanwhile, GSH activated cell proliferation under normal conditions but did not activate cell growth, differentiation, and tissue development under oxidative stress, unlike in normal conditions. In addition, chemokine- and cytokine-related pathways were activated under normal conditions, while the defence systems and cell survival-related pathways such as MAPK, PI3K/AKT, and HIF-1 were mainly activated under oxidative stress. These results suggest that GSH has a different effect depending on the condition, and GSH regulates various cellular processes and enhances liver function under normal conditions, while GSH protects the liver from oxidative stress by activating the defence system of various cells under oxidative stress.

Metallothioneins (MTs) are small cysteine-rich heavy metal-binding proteins that participate in an array of protective metal ion-induced stress responses including oxidative stress [37]. They act as a complementary antioxidant to GSH in cells [38]. Our microarray analysis showed that the GSH-treated groups had downregulated metal ion response-related genes, including *MT* (Figure 4a,d). These results indicated that oxidative stress to HepG2 cells was suppressed by treatment with GSH, resulting in decreased MT expression. It demonstrated that GSH reduced the risk of liver-related diseases associated with oxidative stress.

Angiogenesis is caused by ROS stimulation [39]. Tissue fibrosis is caused by angiogenesis in many organs (including the liver), and blocking angiogenesis may be a promising therapeutic option for patients with advanced fibrosis [40]. GSH treatment inactivated angiogenesis-related terms in this study (Figure 6b). This result demonstrated that oxidative stress to HepG2 cells was suppressed by treatment with GSH, resulting in the inactivation of angiogenesis-related terms. This study demonstrates the potential ability of GSH to prevent fibrosis.

Overall, GSH-treated HepG2 cells showed activation of cellular programmes, including differentiation and development, and enhancement of hepatic functions under normal conditions. In contrast, GSH treatment activated proliferation and antioxidative systems such as MAPK, PI3K/AKT, and HIF-1 in liver cells. We elucidated that GSH alleviated HP’s detrimental effects on cells, such as the overproduction of ROS through the activation of a wide signal around NRF2 in liver cells. The limitation of this research is that there was no validation of transcriptomic analyses for the details but only for antioxidant systems such as HO-1 and GPx. We believe that this study provides important data as a basis for the progress of research on GSH and that the validation of the details obtained from the microarray analysis is required to better understand GSH. In conclusion, to the best of our knowledge, this is the first study comprehensively analysing the molecular mechanisms of GSH in hepatocytes under physiological and stress conditions. These findings support the efficacy of GSH.

## 4. Materials and Methods

### 4.1. Cell Culture and Treatments

HepG2 cells were purchased from the Riken BRC Cell Bank (Tsukuba, Japan). Cells were maintained in Dulbecco’s modified Eagle’s medium (DMEM) (Sigma-Aldrich, Tokyo, Japan) supplemented with 10% foetal bovine serum (Gibco, Massachusetts, USA) and 1% penicillin/streptomycin (Lonza, Japan) at 37 °C in a 5% CO_2_ humidified incubator. The GSH was manufactured by Mitsubishi Corporation Life Sciences Limited (Tokyo, Japan).

The treatment times for GSH and HP were determined by referring to previous research [21,41,42]. The optimal concentrations of each sample were determined by preliminary examination (Supplementary Materials). HepG2 cells were plated on appropriately sized plates for each experiment and incubated for 24 h. The cells were subsequently pretreated with 1 or 2 mM GSH concentrations for 24 h. Subsequently, oxidative stress was induced by adding HP to a final concentration of 2 mM and incubating the cells for 4 h at 37 °C. The effect of GSH treatment on HP-induced oxidative stress was also investigated.

### 4.2. Cell Viability Assay

The cell viability was analysed using the mitochondria-dependent reduction of the MTT assay to determine the effects of GSH on cytotoxicity and antioxidant activity. HepG2 cells were seeded at 5 × 10^3^ cells/well in 96-well plates. Cells were cultured for 24 h at 37 °C and pretreated using 1 or 2 mM GSH and treatment of 2 mM HP (Fujifilm Wako Pure Chemical Co., Tokyo, Japan) as described above, followed by the addition of 10 μL MTT solution to each well, and incubated for 4 h at 37 °C in the dark. One hundred microlitres of 10% sodium dodecyl sulphate (SDS) (Fujifilm Wako Pure Chemical Co., Japan) was added and the mixture was incubated overnight at 37 °C. The optical density (OD) was measured at 570 nm using a Varioskan LUX microplate reader (Thermo Fisher Scientific Co., Ltd., Tokyo, Japan).

### 4.3. ROS Assay

The ROS assay was performed according to the manufacturer’s instructions (Doujindo Co., Ltd., Kumamoto, Japan). HepG2 cells were seeded into black 96-well plates at a density of 5 × 10^3^ cells/well. The cells were pretreated with GSH (1 or 2 mM), then HP was added. After removing the supernatant, cells were washed twice with HBSS. The working solution was added and placed in the incubator for 30 min. After removing the supernatant, the cells were washed with HBSS, and then HBSS was added. The fluorescence intensity of DCF was measured using a Varioskan LUX microplate reader (Thermo Fisher Scientific Co., Ltd., Tokyo, Japan) at 490 nm and 510 nm excitation and emission wavelengths, respectively.

### 4.4. AST, ALT, and Antioxidatant Enzyme Measurements

The concentrations of AST and ALT in the cell supernatant were quantified using ELISA kits according to the manufacturer’s instructions (ab123 and ab234, respectively; Abcam, UK). HepG2 cells were seeded into plates at a density of 1 × 10^5^ cells/well. They were pretreated using 1 or 2 mM GSH and treated with 2 mM HP, followed by collection of the supernatant for storage at −80 °C until analysis. Protein levels in the treated cells were measured using a bicinchoninic acid (BCA) protein assay kit (Thermo Scientific Co., Ltd., Tokyo, Japan), and the AST and ALT levels were adjusted for protein abundance.

The protein expressions of HO-1 and GPx were quantified using ELISA kits according to the manufacturer’s instructions (ab207621 and ab193767, respectively; Abcam, Oregon, UK). The protein level measurement and adjustment were same as for AST and ALT.

### 4.5. RNA Extraction

The total RNA was extracted using ISOGEN (Nippon Gene, Tokyo, Japan). The RNA solution was quantified using a NanoDrop 2000 spectrophotometer (Thermo Fisher Scientific, Tokyo, Japan).

### 4.6. Microarray Experiment

The Clariom S assay system (Thermo Fisher Scientific, Tokyo, Japan) was used for microarray analysis with triplicate RNA samples from each group. Complementary DNA (cDNA) was prepared using the GeneChip WT Plus Reagent kit (Thermo Fisher Science, Tokyo, Japan). The samples were hybridised using a Clariom S GeneChip microarray kit (Thermo Fisher Science, Tokyo, Japan). The CEL data were obtained using GeneChip Scanner 3000 (Thermo Fisher Science, Tokyo, Japan).

### 4.7. Microarray Data Normalisation and Subsequent Analysis

Transcriptome Analysis Console (TAC) software (version 4.0.2, Thermo Fisher Scientific, Waltham, MA, USA) was used to analyse the raw image data obtained by scanning. The raw data were normalised using a robust multichip analysis (SST-RMA) algorithm. Genes meeting the criteria of *p*-value < 0.05 (ANOVA) and FC > 1.1 or FC < −1.1 (in linear space) were considered DEGs.

Further analyses were conducted using the functional annotation tool of the DAVID v6.8 online bioinformatics database to identify enriched GO and KEGG pathways. The DEGs in each BP identified by GO and KEGG analyses were visualised on heat maps using the Morpheus online tool (https://software.broadinstitute.org/morpheus) (accessed 27 July 2023). Functionally clustered modules were identified using the HumanBase public database (https://hb.flatironinstitute.org/) (accessed on 27 July 2023) and analysed using a liver-specific network.

### 4.8. Statistical Analysis

Statistical analyses were performed using EZR version 4.1.2 (Saitama Medical Centre, Saitama, Japan). All data are presented as the mean ± SD. One-way analysis of variance (ANOVA), followed by Dunnett’s multiple comparison test was performed to compare the HP-treated group with the other groups. Statistical significance was defined as * p < 0.05, ** p < 0.01, and *** p < 0.001.

## Data Availability

The supporting data for this article can be found in this paper and in the Supplementary Materials. The microarray data were deposited in the NCBI GEO database and are publicly available (accession number: https://www.ncbi.nlm.nih.gov/geo/query/acc.cgi?acc=GSE242415) (accessed on 4 December 2023).

## Supplementary Materials

The following supporting information can be downloaded from https://www.mdpi.com/article/10.3390/molecules29051090/s1, Figure S1: Cytotoxicity of GSH.
