# Supplementary material for: Comprehensive Transcriptome Profiling of Antioxidant Activities by Glutathione in Human HepG2 Cells
Source: Molecules. 2024 Feb 29;29(5):1090. doi: 10.3390/molecules29051090 (PMC10934103; doi:10.3390/molecules29051090)
Supplement: Supplementary file 1 [file molecules-29-01090-s001.zip › molecules-2811096-supplementary.pdf]

Supplementary Materials

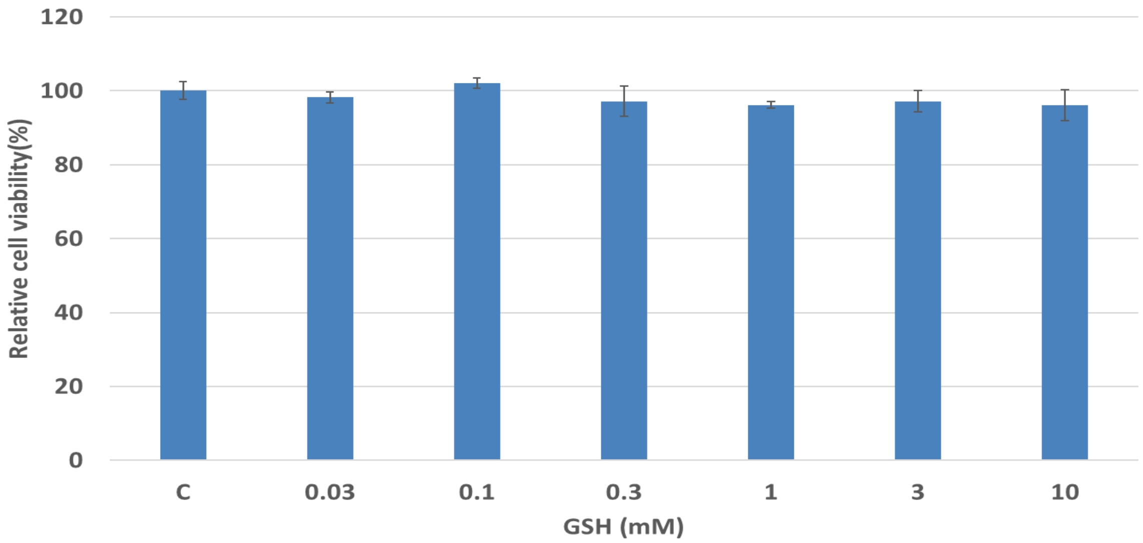

Figure S1. Cytotoxicity of GSH in HepG2. HepG2 cells were treated with GSH for 24h. Cell viability analyzed by MTT assay. All data were presented as the mean ± SD. Comparison with control (C) were carried out using a one-way analysis of variance (ANOVA) followed by Dunnett's test.
